# Old Drug, New Science: Metformin and the Future of Pharmaceutics

**DOI:** 10.3390/pharmaceutics18010077

**Published:** 2026-01-07

**Authors:** Alfredo Caturano, Davide Nilo, Roberto Nilo, Marta Chiara Sircana, Enes Erul, Katarzyna Zielińska, Vincenzo Russo, Erica Santonastaso, Ferdinando Carlo Sasso

**Affiliations:** 1 Department of Human Sciences and Promotion of the Quality of Life, San Raffaele Roma University, 00166 Rome, Italy; 2 Department of Advanced Medical and Surgical Sciences, University of Campania “Luigi Vanvitelli”, 80138 Naples, Italy; nilodavide@gmail.com (D.N.);; 3 Data Collection G-STeP Research Core Facility, Fondazione Policlinico Universitario A. Gemelli IRCCS, 00168 Roma, Italy; 4 Department of Medical, Surgical and Pharmacology, University of Sassari, 07100 Sassari, Italy; 5 Department of Medical Oncology, Ankara University Faculty of Medicine, 06620 Ankara, Turkey; eneserul@hotmail.com; 6 Scanmed Medical Center in Krakow, 30150 Krakow, Poland; 7 Department of Biology, College of Science and Technology, Sbarro Institute for Cancer Research and Molecular Medicine, Temple University, Philadelphia, PA 19122, USA; 8 Division of Cardiology, Department of Medical Translational Sciences, University of Campania “Luigi Vanvitelli”, 80138 Naples, Italy; 9 Independent Researcher, 81024 Maddaloni, Italy

**Keywords:** metformin, pharmaceutics, drug delivery, pharmacogenetics, gut-liver axis, personalized medicine, formulation science, translational pharmacology

## Abstract

Metformin, a 60-year-old biguanide and cornerstone of type 2 diabetes therapy, continues to challenge and inspire modern pharmaceutical science. Despite its chemical simplicity, metformin displays highly complex pharmacokinetic and pharmacodynamic behavior driven by transporter dependence, luminal activity, and formulation-sensitive exposure. Originally regarded as limited by low permeability and incomplete absorption, metformin has emerged as a paradigm for gut-targeted therapy, controlled- and delayed-release systems, and personalized pharmaceutics. Growing evidence has repositioned the intestine, rather than systemic plasma exposure, as a major site of action, highlighting the central role of organic cation transporters and multidrug efflux systems in determining efficacy, variability, and gastrointestinal tolerability. Beyond metabolic control, insights into transporter regulation, pharmacogenetics, microbiome interactions, and manufacturing quality have expanded metformin’s relevance as a model compound for contemporary drug development. Advances in formulation design, quality-by-design manufacturing, and regulatory control have further reinforced its clinical robustness, while repurposing efforts in oncology, immunometabolism, and regenerative medicine underscore its translational potential. This review integrates mechanistic pharmacology, formulation science, and clinical translation to position metformin not merely as an antidiabetic agent, but as a didactic model illustrating the evolution of pharmaceutics from molecule-centered design to system-oriented, precision-driven therapy.

## 1. Introduction

Few drugs in the history of medicine have maintained their centrality as tenaciously as metformin. Introduced more than 60 years ago as an oral antidiabetic agent, metformin remains the undisputed first-line therapy for type 2 diabetes and one of the most prescribed medications worldwide [1]. Its safety, low cost, and broad metabolic effects have made it an enduring cornerstone of clinical practice [2]. Yet, paradoxically, this familiarity often obscures how much remains to be learned about this deceptively simple molecule.

**Figure 1:**
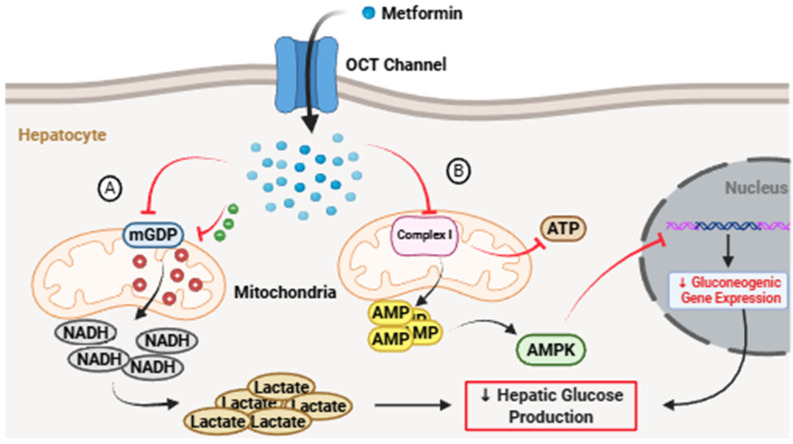
Mechanisms of metformin action on hepatic gluconeogenesis. (**A**) Mitochondrial mGPD inhibition: Metformin targets mitochondrial glycerol-3-phosphate dehydrogenase (mGPD), suppressing the glycerophosphate shuttle. The resulting increase in the cytosolic NADH/NAD^+^ ratio creates a reductive environment that prevents the conversion of non-carbohydrate precursors (lactate and glycerol) into glucose. (**B**) Complex I and AMP signaling: By inhibiting Mitochondrial Complex I, metformin reduces ATP synthesis and elevates intracellular AMP levels. This metabolic shift activates AMPK and antagonizes cAMP signaling, leading to the transcriptional downregulation of key gluconeogenic enzymes.

Metformin is a small, highly hydrophilic biguanide whose chemical structure consists of two guanidine groups linked by a dimethyl-substituted amine. This simple molecular architecture confers high aqueous solubility, negligible plasma protein binding, and almost no passive membrane permeability, placing the drug in Biopharmaceutics Classification System (BCS) class III. From a biopharmaceutic standpoint, metformin is a prototypical Biopharmaceutics Classification System (BCS) class III compound, characterized by high solubility but low intestinal permeability [3,4]. As a consequence, its absorption is inefficient, saturable, and entirely dependent on carrier-mediated transport rather than passive diffusion [5]. These physicochemical features, far from being a limitation alone, have made metformin an instructive model for studying the interplay between drug chemistry, transporter biology, and formulation science. Pharmacologically, metformin lowers glucose primarily by suppressing hepatic gluconeogenesis, an effect mediated through modulation of mitochondrial respiratory chain complex I, changes in cellular energy charge, and activation of AMP-activated protein kinase (AMPK) (Figure 1) [6,7]. However, it is now clear that metformin’s actions extend well beyond the liver. The drug accumulates in the intestinal lumen at concentrations several hundred-fold higher than those observed in plasma, where it influences glucose absorption, bile acid metabolism, incretin secretion, and gut–brain signaling [8,9]. These luminal and epithelial effects are increasingly recognized as central to its therapeutic efficacy and tolerability. In parallel, metformin interacts with the gut microbiota, promoting shifts in microbial composition and function that contribute to improved metabolic homeostasis. This combination of transporter dependence, luminal activity, and microbiome modulation distinguishes metformin from most orally administered small molecules [10] and explains its continued relevance as a model compound in modern pharmaceutics.

**Figure 2:**
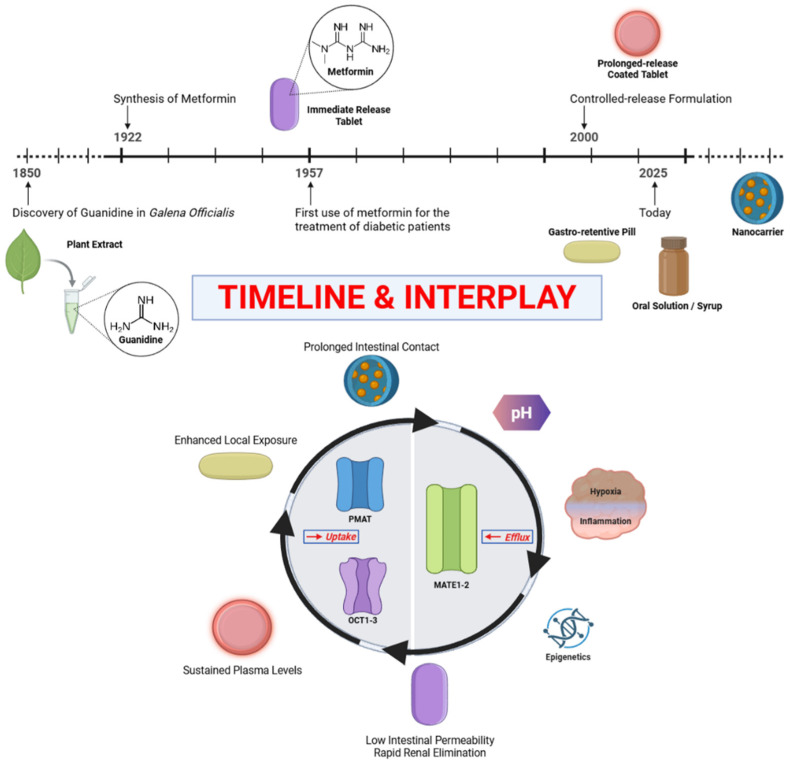
The evolving pharmaceutic landscape of metformin. The upper panel illustrates the historical and technological evolution of metformin, from the identification of guanidine-containing plant extracts (*Galega officinalis*) and chemical synthesis of biguanides (1922), to first clinical use in diabetes (1957), large-scale outcome validation (UKPDS, late 1990s), and the progressive development of advanced oral formulations, including extended-release, delayed-release, gastro-retentive systems, and emerging nanocarrier-based approaches. The lower panel depicts the conceptual interplay between formulation strategies and transporter-mediated pharmacokinetics that defines metformin’s modern pharmaceutic behavior. Low intrinsic intestinal permeability and rapid renal elimination limit systemic exposure, while uptake transporters (OCT1–3, PMAT) and efflux transporters (MATE1/2-K) regulate tissue-specific accumulation. Formulation-driven prolongation of intestinal residence time enhances local exposure, whereas environmental and biological modifiers (including luminal pH, hypoxia, inflammation, and epigenetic regulation of transporters) further influence absorption, tolerability, and efficacy. Together, this schematic highlights how metformin has transitioned from a conventional oral drug to a model compound for transporter-informed and gut-targeted pharmaceutic design.

Metformin’s clinical story is deeply rooted in medical history (Figure 2). The biguanide scaffold was first synthesized in 1922 by Werner and Bell during investigations into substituted guanidines, although no immediate therapeutic application followed [11]. Interest in biguanides was later revived through observations of the glucose-lowering properties of *Galega officinalis*, a medicinal plant rich in guanidine derivatives [12,13]. Building on this background, the French physician Jean Sterne reported the first clinical use of dimethylbiguanide (metformin) as an oral antidiabetic agent in 1957, demonstrating a favorable benefit–risk profile compared with other biguanides available at the time [14]. Based on these findings, metformin was first marketed in France in 1959 by Laboratoires Aron under the name *Glucophage*, a term reflecting its glucose-lowering action. This historical trajectory, from early chemical synthesis to botanical observation and finally clinical validation, underscores how metformin emerged not from rational drug design, but from iterative pharmacological insight [12].

Despite decades of use, metformin continues to intrigue researchers because many of its pharmacological mechanisms are only partially understood. Its pleiotropic benefits—ranging from glycemic control to cardiovascular protection, anti-inflammatory actions, and even anti-aging effects—challenge the notion that an old drug has little to offer in the age of nanomedicine and precision pharmacology [15].

In this review, we summarize the evolving understanding of metformin pharmacology, with particular emphasis on intestinal mechanisms, transporter biology, and emerging formulation strategies. Unlike recent reviews that focus on these elements in isolation, the present work adopts an integrated pharmaceutics-oriented perspective, linking transporter function with formulation design, manufacturing and quality considerations, pharmacogenetic variability, and translational repurposing applications, thereby positioning metformin as a model compound for contemporary drug development.

## 2. Lessons from the Past: Pharmacokinetics and Biopharmaceutics

Metformin’s chemical simplicity—an unadorned biguanide—belies its complex pharmacokinetic behavior. The molecule is hydrophilic, non-lipophilic, and virtually non-metabolized, characteristics that explain both its safety and its biopharmaceutical limitations. Its oral bioavailability averages only 50–60%, and absorption occurs primarily in the small intestine through active transport mechanisms mediated by organic cation transporters (OCT1, OCT2, OCT3) [15,16,17]. From a quantitative pharmacokinetic perspective, immediate-release (IR) metformin formulations typically reach peak plasma concentrations (Cmax) of approximately 1.5–3.0 μg/mL following standard oral doses (500–1000 mg), with a median time to peak concentration (Tmax) of 2–3 h. In contrast, extended-release (XR) formulations are characterized by a delayed Tmax, usually ranging between 6 and 8 h, and a lower peak concentration, with Cmax values reduced by approximately 20–30% compared with IR formulations, while maintaining comparable overall exposure (AUC). Absolute oral bioavailability remains similar across formulations (approximately 50–60%), indicating that modified-release technologies primarily modulate the rate, rather than the extent, of absorption [18,19]. These differences translate into smoother plasma concentration–time profiles for XR formulations, which are associated with improved gastrointestinal tolerability and adherence, despite equivalent glycemic efficacy.

Unlike most small molecules, metformin does not rely on passive diffusion across biological membranes. Its uptake is transporter-dependent, tissue-specific, and saturable, producing a nonlinear relationship between dose and plasma concentration [20]. This property has been an obstacle for classical controlled-release systems and a challenge for dose optimization in patients with variable renal or hepatic function [17,20]. Moreover, metformin undergoes no hepatic metabolism and is excreted unchanged in urine through the action of multidrug and toxin extrusion transporters (MATE1 and MATE2-K) [21]. Consequently, pharmacokinetics are heavily influenced by renal clearance and transporter activity rather than by enzyme metabolism [21]. These features made metformin an early paradigm of the “biopharmaceutics classification system” conundrum—its high solubility but low permeability classifies it as a BCS class III compound, limiting oral absorption and complicating sustained-release formulation strategies [22]. Historically, these limitations encouraged the development of simple immediate-release tablets administered two or three times daily. For decades, pharmaceutical innovation around metformin was constrained by its chemical and absorption properties. Yet those same constraints have since become the springboard for exploring new delivery paradigms, demonstrating how biopharmaceutic challenges can stimulate creativity rather than end it.

Early formulation efforts sought not only to improve metformin’s absorption profile but also to integrate it with complementary glucose-lowering agents in fixed-dose oral combinations. As its role as a foundational therapy became established, metformin was co-formulated with sulfonylureas, thiazolidinediones, DPP-4 inhibitors, and later SGLT2 inhibitors to simplify regimens and enhance adherence [23]. These combinations were particularly relevant given metformin’s BCS class III properties: co-formulation required careful control of dissolution, excipient compatibility, and tablet integrity to preserve the bioavailability of both components [24]. Parallel to these developments, several dosage form innovations were explored to overcome the drug’s short intestinal absorption window and transporter-dependent uptake, including buffered tablets, polymer-based sustained-release matrices, osmotic pump tablets, and early gastro-retentive prototypes [25,26]. Although many of these initial approaches offered only incremental improvements, they laid the foundation for the more sophisticated delivery systems discussed in later sections and highlighted how metformin’s biopharmaceutical constraints directly shaped formulation design.

## 3. The Gut–Liver Axis and the Rebirth of Metformin Research

A striking shift in the understanding of metformin occurred in the last decade with the realization that the gut, not the systemic circulation, may be the principal site of action [27]. Accumulating evidence suggests that metformin’s primary metabolic effects are initiated within the intestinal wall, influencing glucose absorption, bile acid turnover, and the secretion of gut hormones such as GLP-1 [8]. This paradigm reframes earlier pharmacokinetic observations: low systemic bioavailability and limited plasma exposure may not reflect therapeutic inefficiency, but rather a deliberate pharmacologic feature. Indeed, luminal concentrations of metformin can exceed plasma levels by more than 300-fold, creating a direct interface with intestinal epithelial cells, enteroendocrine signaling pathways, and the gut microbiota [10].

Beyond local metabolic effects, metformin appears to influence the gut–brain axis, a bidirectional communication network linking intestinal nutrient sensing to central metabolic regulation [28]. Recent studies suggest that metformin modulates enteroendocrine cell activity and vagal afferent signaling, thereby influencing central appetite control, hepatic glucose production, and systemic insulin sensitivity [28,29]. One of the molecular mediators implicated in this process is Rap1, a small GTPase involved in intracellular signaling downstream of cAMP and incretin receptors. Experimental models suggest that metformin may indirectly influence Rap1-related signaling in intestinal and neural tissues, integrating nutrient sensing with neuroendocrine responses. Through this pathway, metformin may attenuate hepatic gluconeogenesis not only via direct hepatic AMPK activation, but also through gut-derived neural and hormonal signals that converge on central regulatory circuits [30].

The gut microbiota further amplifies this gut–brain–liver interplay. A randomized trial has demonstrated that metformin reshapes microbial composition, enhancing short-chain fatty acid-producing species and modulating bile acid pools, which may partly mediate its systemic metabolic effects [31]. These microbiota-derived metabolites influence enteroendocrine secretion, intestinal barrier function, and vagal signaling, providing an additional layer through which metformin may engage Rap1-dependent and incretin-mediated pathways [32,33,34]. Collectively, these findings position the gut microbiome as an active intermediary rather than a passive bystander in metformin pharmacology.

From a pharmaceutic standpoint, this gut-centered model opens entirely new horizons. If the intestine is indeed a major target, formulations designed to maximize local exposure while minimizing systemic absorption could enhance efficacy and tolerability [35]. Such approaches are now being explored with delayed-release metformin, engineered to dissolve at higher pH in the distal small intestine, thereby reducing gastrointestinal side effects while preserving glycemic control [36]. This shift also situates metformin at the intersection of drug delivery and systems biology. It challenges formulation scientists to think not only in terms of plasma levels but also in terms of tissue-specific exposure and microenvironmental modulation, a concept that is rapidly reshaping modern pharmaceutics.

## 4. Formulation Innovations: From Sustained Release to Nanotechnology

The effort to refine metformin delivery has produced an impressive variety of technological approaches, each reflecting the evolution of pharmaceutical science itself (Table 1).

### 4.1. Transporter-Informed Formulation Design

Beyond conventional pharmacokinetic optimization, contemporary formulation strategies increasingly recognize transporter biology as a dynamic determinant of metformin efficacy and tolerability. Intestinal uptake transporters, including OCT1, OCT3, and PMAT, govern epithelial entry of metformin, while efflux transporters such as MATE1 and MATE2-K regulate intracellular residence time and luminal re-exposure [37,38,39,40]. Importantly, the activity of these transporters is not static but influenced by luminal pH, local drug concentration, inflammation, and formulation-driven residence time. Rapid release and high local concentrations may transiently saturate uptake transporters while enhancing efflux, contributing to gastrointestinal intolerance, whereas controlled or delayed-release formulations may favor more homogeneous epithelial exposure and improved tolerability [15,19,37,38,39].

Formulation design therefore directly intersects with transporter regulation. Gastro-retentive and extended-release systems aim to align drug release with regions of higher transporter expression, while delayed-release formulations shift delivery toward distal intestinal segments to modulate local exposure without excessive systemic absorption [40]. These approaches exemplify how pharmaceutics can leverage transporter-mediated processes rather than merely accommodate them, transforming metformin’s perceived biopharmaceutical limitations into therapeutic opportunities. In parallel, growing evidence highlights the role of the gut microbiota as an additional, formulation-sensitive modulator of metformin response. Metformin has been shown to increase the abundance of *Akkermansia muciniphila*, a mucin-degrading bacterium associated with improved gut barrier integrity, reduced inflammation, and enhanced metabolic control. This microbial shift appears to depend not only on drug presence but also on luminal concentration gradients and intestinal exposure time, both of which are influenced by formulation strategy [40]. By shaping microbial composition and bile acid metabolism, metformin–microbiome interactions may indirectly affect enteroendocrine signaling, transporter expression, and gastrointestinal tolerability [41].

The recent detection of N-nitrosodimethylamine (NDMA) and related nitrosamine impurities in metformin products has highlighted the importance of pharmaceutical quality control throughout the manufacturing process [42]. NDMA formation is not intrinsic to metformin itself but may arise from the presence of dimethylamine precursors, nitrosating agents, contaminated solvents, or degradation pathways during synthesis and storage [43]. In response, regulatory agencies including the U.S. Food and Drug Administration (FDA) and the European Medicines Agency have issued guidance requiring comprehensive risk assessment, validated analytical testing, and strict control of manufacturing conditions in accordance with ICH M7 principles. Current strategies to mitigate nitrosamine risk include optimization of synthetic routes, tighter specifications for raw materials, avoidance of secondary amines and nitrosating reagents, improved solvent quality, and enhanced stability monitoring [44,45,46].

Importantly, ongoing regulatory surveillance and post-marketing testing have ensured that commercially available metformin products now comply with acceptable intake limits for nitrosamines. This episode underscores how modern pharmaceutics extends beyond delivery optimization to encompass manufacturing robustness, quality-by-design, and regulatory vigilance, reinforcing patient safety without altering the fundamental therapeutic role of metformin.

### 4.2. Manufacturing Quality and Nitrosamine Risk Control

On the manufacturing side, one of the most relevant advances for metformin has been the optimization of large-scale API synthesis and purification to improve yield, reproducibility, and impurity control. Industrial routes typically rely on the condensation of a dimethylamine salt (commonly dimethylammonium chloride) with dicyandiamide to form metformin, followed by conversion to the hydrochloride salt and controlled crystallization. Recent process innovations have focused on improving conversion efficiency and downstream purification while limiting residual dimethylamine (DMA), a key precursor of nitrosamines. In this context, WO2019154769A1 describes an improved manufacturing process for metformin hydrochloride aimed at scalable production with tighter control of reaction-related impurities and consistent solid-state quality [47].

From a regulatory perspective, mitigation of DMA/NDMA risk is now managed through a quality-by-design framework that includes (i) risk assessment of raw materials and reagents that can introduce secondary amines or nitrosating species, (ii) validated analytical testing for nitrosamines, and (iii) defined process controls and specifications designed to prevent formation and carryover of nitrosamine impurities. FDA’s current guidance on nitrosamine control outlines these expectations for both API and finished products, supporting routine surveillance and corrective actions when needed. Consistent with this approach, FDA and EMA communications on metformin note an acceptable daily intake benchmark for NDMA and emphasize ongoing monitoring to ensure marketed products remain within acceptable limits [45,46].

Early innovations centered on extended-release and controlled-release tablets designed to smooth plasma concentration profiles, reduce dosing frequency, and improve gastrointestinal tolerability [48]. The introduction of hydrophilic polymer matrices (such as hydroxypropyl methylcellulose) and osmotic delivery systems has led to a reduction in gastrointestinal side effects, thereby improving therapeutic adherence [49]. However, the next generation of formulations aims beyond pharmacokinetic convenience. Gastro-retentive systems, which prolong the residence time of the tablet in the stomach or upper intestine, seek to exploit the region where transporters are most expressed [50]. Mucoadhesive formulations, floating systems, and expandable polymers are among the strategies used to localize the drug in the upper gastrointestinal tract [51]. At the micro- and nanoscale, nanocarriers, liposomes, and polymeric nanoparticles have been tested to enhance the permeability and controlled release of metformin [52]. Although the molecule’s hydrophilicity presents challenges for encapsulation, innovative approaches-such as ionic-gelation nanoparticles or solid-lipid carriers-have shown promise in preclinical studies [53,54]. These systems may not only improve bioavailability but also enable targeted co-delivery with complementary agents, such as antioxidants or anti-inflammatory compounds, to exploit metformin’s pleiotropic effects [55,56,57]. Still, the field must balance innovation with pragmatism. Over-engineering a molecule with proven safety and simplicity risks undermining one of its strengths: cost-effectiveness. The challenge for pharmaceutics is therefore not to reinvent metformin, but to adapt formulation science to maximize its potential where it matters most—local targeting, controlled exposure, and patient adherence. Metformin thus serves as a model molecule for the rational design of delivery systems for hydrophilic drugs, highlighting the interplay between transporter pharmacology, material science, and patient-centered formulation. This progressive evolution of formulation, from conventional tablets to transporter-targeted and nanotechnology-based systems, is summarized in Figure 1.

From classical oral formulations to modern nanocarrier and gut-targeted systems, advances in pharmaceutics aim to optimize drug exposure at the intestinal, hepatic, and tumor levels. Transporter biology (OCTs, PMAT, MATEs) now bridges formulation science with precision therapy, illustrating how metformin has evolved from a glucose-lowering agent to a model for translational pharmaceutics.

## 5. Pharmacogenetics and Personalized Pharmaceutics

The next frontier in metformin research lies in understanding individual variability in response [58]. Despite its widespread use, up to 30–35% of patients exhibit suboptimal glycemic control or intolerance [59,60]. Genetic differences in drug transporters—particularly loss-of-function variants of OCT1—have been implicated in reduced hepatic uptake and diminished efficacy [61]. Similarly, polymorphisms in MATE1 and MATE2-K influence renal elimination and systemic exposure [62].

These observations place metformin at the center of ongoing debates in precision medicine, where variability in drug response remains a major unresolved challenge [63]. While pharmacogenetic testing has been widely discussed, its integration into routine clinical decision-making remains limited for most drugs, often due to narrow therapeutic windows, complex metabolism, or lack of actionable formulation alternatives [64]. Metformin represents a notable exception. Its wide safety margin, transporter-dependent disposition, and availability of multiple formulation strategies make it uniquely suited as a test case for translating pharmacogenetic insight into practical pharmaceutical solutions [2,15,65].

Against this backdrop, pharmacogenetic profiling could therefore inform not only dosing but also formulation selection [66]. Patients with reduced hepatic transport capacity might benefit more from gut-targeted or delayed-release formulations that emphasize local mechanisms, whereas those with normal transporter activity could rely on conventional extended-release tablets [67]. This approach marks a conceptual shift toward personalized pharmaceutics, where formulation is tailored not merely to disease but to the individual’s genetic and physiological context. The integration of in silico modeling, physiologically based pharmacokinetics (PBPK), and AI-driven optimization could further refine these predictions, enabling drug–device combinations that adapt to patient-specific absorption and elimination profiles [68,69,70,71].

Metformin provides an ideal proving ground for such innovations because its safety margin is wide, its pharmacology well characterized, and its clinical outcomes easily measurable. In this sense, personalized pharmaceutics may finally fulfill the promise of precision medicine–not by inventing new drugs, but by intelligently redesigning old ones.

## 6. Clinical Perspectives and Translational Relevance

More than six decades of clinical use have made metformin one of the best-characterized oral antidiabetic agents, with solid pharmacologic and pharmaceutic data linking formulation design to therapeutic performance. In patients with type 2 diabetes, metformin consistently reduces HbA1c by approximately 1.0–1.5%, independently of baseline glycemia, and decreases the risk of diabetes-related endpoints by 30–35%, as demonstrated in the UK Prospective Diabetes Study (UKPDS) and its long-term follow-up [1,72,73]. These benefits persist despite the drug’s low bioavailability (50–60%) and lack of hepatic metabolism, underscoring the efficiency of transporter-mediated absorption and distribution [16].

Comparative trials show that extended-release and gastro-retentive formulations achieve similar glycemic control to immediate-release tablets but with 20–30% lower rates of gastrointestinal side effects and better adherence [22,50]. In a meta-analysis of 2609 patients from nine randomized clinical trials, extended-release metformin reported reduced gastrointestinal intolerance without compromising efficacy [48]. Moreover, delayed-release intestinal formulation achieved comparable HbA1c reduction (0.5%) compared to the extended-release (0.7%) [48].

Clinical pharmacogenetic data provide further granularity. Loss-of-function variants of OCT1 (SLC22A1) are associated with a 20–30% attenuation in HbA1c response, while MATE1 and MATE2-K polymorphisms influence metformin’s plasma exposure and gastrointestinal tolerability [61,62]. These findings suggest that genotype-informed formulation or dosing adjustments could optimize outcomes, a concept already being explored in pharmacogenomic implementation studies [66]. Beyond glucose lowering, observational cohorts and meta-analyses have linked metformin use to 20–25% reductions in cardiovascular events, lower cancer incidence (notably in colorectal, breast, and prostate malignancies), and delayed cognitive decline [72,73,74,75,76]. Although causality remains debated, these effects may partly arise from tissue-specific transporter expression and local AMPK activation and mitochondrial function [77]. Collectively, clinical data reaffirm metformin as a model compound in which pharmaceutic design, transporter biology, and therapeutic benefit converge, illustrating the full translational cycle from formulation to clinical precision [78].

In the contemporary therapeutic landscape, metformin continues to occupy a central position despite the rapid expansion of novel glucose-lowering agents. Current international guidelines consistently recommend metformin as first-line therapy for type 2 diabetes, primarily due to its efficacy, long-term safety, affordability, and cardiovascular neutrality or benefit [79]. While newer drug classes, including glucagon-like peptide-1 receptor agonists and sodium–glucose cotransporter-2 inhibitors, have demonstrated additional benefits in weight reduction, cardiovascular protection, and renal outcomes, their higher cost and specific safety considerations limit universal first-line adoption [79,80,81,82]. Consequently, metformin remains the foundation upon which these agents are added, rather than replaced, in most treatment algorithms. This positioning highlights how metformin’s clinical and pharmaceutic versatility continues to complement, rather than compete with, newer therapies in real-world practice.

Despite its favorable benefit–risk profile, metformin therapy is associated with several well-recognized limitations. Gastrointestinal intolerance, including diarrhea, nausea, and abdominal discomfort, represents the most common adverse effect and the leading cause of treatment discontinuation, particularly with immediate-release formulations [83]. Long-term metformin use has also been associated with reduced vitamin B12 absorption, which may contribute to anemia or peripheral neuropathy in susceptible individuals [84]. In addition, metformin monotherapy may be insufficient in patients with long-standing or advanced type 2 diabetes, necessitating early combination therapy [85]. Finally, caution is required in elderly and frail patients and in those with impaired renal, hepatic, or cardiopulmonary function, where altered drug clearance or intercurrent illness may increase the risk of adverse outcomes [86].

Beyond these relatively frequent and generally manageable limitations, a distinct and far less common concern is the risk of metformin-associated lactic acidosis (MALA), particularly in elderly patients with moderate to severe renal impairment. However, the incidence of MALA has markedly declined in recent years, largely due to eGFR-based dosing guidelines, routine renal function monitoring, and the discontinuation of metformin during acute illnesses. MALA typically arises in the context of severe renal failure (eGFR < 30 mL/min/1.73 m^2^), hypovolemia, or tissue hypoxia (e.g., sepsis, cardiogenic shock, stroke), emphasizing the need to optimize both drug handling and formulation safety in frail or acutely ill individuals [60,87,88]. Contemporary evidence indicates that MALA occurs almost exclusively when metformin is continued during acute intercurrent illnesses, such as sepsis, dehydration, vomiting, or diarrhea, that transiently impair renal clearance and promote drug accumulation. Since metformin is eliminated almost entirely through glomerular filtration and tubular secretion, even short-term kidney dysfunction can markedly increase plasma concentrations [89]. In severe presentations, characterized by metabolic acidosis and elevated lactate levels, continuous renal replacement therapy represents the treatment of choice; extracorporeal elimination is generally recommended for lactate > 20 mmol/L or pH ≤ 7.0 [90].

In patients with chronic kidney disease (eGFR > 30 mL/min/1.73 m^2^), therapeutic optimization can be achieved through combinatorial regimens, using low-dose and/or delayed-release metformin in association with other glucose-lowering agents (such as DPP-4 inhibitors, SGLT2 inhibitors, or GLP-1 receptor agonists) to maintain glycemic efficacy while minimizing systemic accumulation [91,92]. This de-intensified strategy embodies the essence of personalized pharmaceutics: maximizing therapeutic benefit while mitigating risk through rational dose design and formulation innovation.

## 7. Broader Horizons: Repurposing and Translational Opportunities

From a pharmaceutical perspective, such repurposing efforts invite targeted delivery systems capable of achieving therapeutic concentrations in specific tissues—tumors, liver, or even the central nervous system—without systemic overload [15].

### 7.1. Metabolic and Endocrine Repurposing

Clinically, the interest in repurposing metformin stems from its pleiotropic metabolic and anti-inflammatory actions, which extend beyond type 2 diabetes. The drug is also employed as adjuvant therapy in type 1 diabetes with insulin resistance, where it can reduce insulin requirements and body weight [93]; in metabolic dysfunction-associated steatotic liver disease and cirrhosis, improving insulin sensitivity and modestly lowering transaminases and hepatic fat [94]; and in polycystic ovary syndrome, where it enhances ovulation, menstrual regularity, and the overall metabolic profile [95].

### 7.2. Immunometabolic and Regenerative Applications

In addition to metabolic and endocrine indications, emerging preclinical evidence suggests a role for metformin in tissue repair and neuroregeneration. In a rat model of sciatic nerve injury, metformin treatment accelerated functional recovery, enhanced axonal regeneration and remyelination, and promoted a phenotypic shift in macrophages toward a pro-regenerative M2 profile. Mechanistically, these effects were mediated by activation of the AMPK/PGC-1α/PPAR-γ signaling axis, as pharmacological inhibition of AMPK abolished metformin-induced M2 polarization [96]. These findings highlight metformin’s capacity to modulate immunometabolic pathways beyond glucose control, supporting its broader repurposing potential in inflammatory and regenerative conditions.

### 7.3. Oncology: Mechanistic Rationale and Translational Limits

Beyond formulation-based innovation, several metformin derivatives and biguanide-related compounds have been developed to enhance potency, tissue targeting, or mechanistic specificity [15]. Chemical modification strategies have aimed to improve mitochondrial targeting, increase lipophilicity, or amplify AMPK-dependent effects, particularly in oncology and metabolic disease models [97]. To date, most metformin derivatives have demonstrated promising preclinical activity, especially in cancer cell lines and animal models, where enhanced intracellular accumulation and mitochondrial inhibition have been observed [98]. However, translation into clinical practice has been limited. Earlier biguanide analogues such as phenformin were withdrawn due to unacceptable safety profiles, underscoring the delicate balance between efficacy and toxicity within this chemical class. More recent derivative approaches, including mitochondria-targeted or conjugated biguanides, remain largely investigational, with clinical evaluation still in early phases [99]. At present, no metformin derivative has demonstrated clear clinical superiority over the parent compound. These efforts nevertheless provide valuable insight into structure–activity relationships and reinforce the concept that metformin itself represents an optimal balance of safety, efficacy, and affordability [100]. From a pharmaceutic perspective, these findings further justify prioritizing formulation innovation and targeted delivery over molecular modification.

These largely off-label uses exemplify metformin’s capacity to modulate systemic energy metabolism and inflammation. For pharmaceutics, they highlight how rational formulation design and drug repurposing can extend the clinical life cycle of a molecule with an exceptional safety record and multifaceted biological profile. Novel carriers, including mitochondria-targeted prodrugs, polymeric conjugates, and nanoparticle-based co-delivery systems, are being explored to exploit these broader biochemical properties while overcoming its limited permeability [101,102].

Among repurposing indications, oncology has attracted particular attention due to metformin’s ability to modulate cellular energy metabolism, mitochondrial function, and tumor-associated signaling pathways. At the molecular level, metformin inhibits mitochondrial respiratory chain complex I, leading to reduced ATP production and increased AMP/ATP ratios, thereby activating AMPK. AMPK activation suppresses anabolic pathways, inhibits mTOR signaling, and limits tumor cell proliferation under conditions of metabolic stress [4,97]. Beyond direct effects on cancer cells, metformin has been shown to influence the tumor microenvironment. By lowering systemic insulin levels and modulating inflammatory signaling, metformin may indirectly reduce growth-promoting cues within the tumor microenvironment [4,97]. Experimental evidence also suggests that metformin can enhance antitumor immune responses by improving CD8^+^ T-cell metabolic fitness and reducing immunosuppressive myeloid populations, thereby sensitizing tumors to immune checkpoint inhibition [103].

Importantly, the anticancer activity of metformin appears highly context-dependent and influenced by transporter expression [104]. Uptake via OCTs and PMAT is required for intracellular accumulation, and differential transporter expression across tumor types may partly explain heterogeneous clinical outcomes [104,105]. From a pharmaceutic perspective, these observations reinforce the rationale for targeted delivery strategies, including tumor-directed formulations or combination regimens, to overcome limited permeability and maximize local exposure. Although epidemiological studies and retrospective analyses suggest reduced cancer incidence and improved outcomes in metformin-treated patients, prospective randomized trials, though still limited, have yielded mixed results [74]. Lung cancer represents one of the most extensively studied oncologic contexts for metformin repurposing. Retrospective clinical analyses indicate that metformin use in patients with type 2 diabetes and lung cancer is associated with improvements in distant metastasis–free survival, progression-free survival, and overall survival [106,107,108]. These effects have been linked to modulation of metabolic and growth-related pathways, including inhibition of the AMPK/LKB1/mTOR axis, attenuation of insulin and insulin-like growth factor-1 signaling, and induction of apoptosis and autophagy. Given the central role of metabolic reprogramming in lung cancer progression, metformin provides a biologically plausible adjunctive strategy [106,107,108]. However, reported clinical outcomes remain heterogeneous, underscoring the need for biomarker-guided and formulation-aware approaches to clarify its therapeutic role. Collectively, these findings support a strong mechanistic rationale for metformin in oncology while underscoring the need for formulation-aware and biomarker-guided approaches to translate preclinical promise into clinical benefit.

Repurposing also exemplifies a sustainable model of drug innovation. Instead of focusing exclusively on new molecular entities, the field of pharmaceutics can derive substantial impact by re-engineering existing molecules with proven safety records. In this framework, metformin becomes not just a drug, but a platform for translational formulation research, bridging basic pharmacology with advanced materials science.

## 8. Transporters: The Missing Link in Metformin’s Translational Pharmacology

Recent evidence continues to reshape the understanding of metformin’s role beyond metabolism and glucose homeostasis. Metformin’s variable anticancer effects has been suggested to depend on the expression and regulation of its solute carrier transporters, which govern cellular uptake and efflux [109]. The interplay between influx transporters (OCT1-3, PMAT) and efflux systems (MATE1-2) determines intracellular accumulation and, consequently, therapeutic efficacy [109]. These transporters are themselves subject to epigenetic modulation, transcriptional control, and environmental influences such as hypoxia and acidic pH within the tumor microenvironment [110,111]. Collectively, these insights identify metformin transport as a potential determinant and modifiable node of its pharmacologic activity. For pharmaceutics, this perspective reinforces how transporter biology bridges drug formulation, tissue targeting, and precision oncology, opening new avenues for metformin-based delivery and combination strategies. A practical example of this framework is provided by oncology. Tumor cells expressing high levels of OCT3 or PMAT can accumulate metformin intracellularly, enabling mitochondrial complex I inhibition and AMPK activation, whereas tumors with low transporter expression may remain pharmacologically insensitive despite adequate systemic exposure [110,112]. Similarly, hypoxic and acidic tumor microenvironments can alter transporter activity, further modulating intracellular drug levels [113]. From a pharmaceutic perspective, these observations explain why systemic dosing alone is insufficient to predict response and why transporter-aware formulation or tumor-targeted delivery strategies are required to translate mechanistic promise into clinical efficacy.

An overview of the principal metformin transporters, their regulatory mechanisms, and pharmaceutic implications is provided in Table 2.

## 9. Conclusions: The Pharmaceutics of Longevity

The enduring story of metformin mirrors the trajectory of pharmaceutics itself. Both began with simple formulations and empirical observations; both evolved toward mechanistic sophistication and technological refinement. Metformin’s journey—from a modest biguanide tablet to a testbed for transporter biology, microbiome research, and nanocarrier design—demonstrates how the science of drug delivery shapes the meaning and destiny of a therapeutic molecule. For pharmaceutics as a discipline, metformin is more than a legacy drug. It is a didactic model that encapsulates every core concept of our field: absorption, distribution, transport, metabolism, variability, and targeted delivery. Its longevity underscores that pharmaceutical innovation does not necessarily depend on novelty, but on our ability to reinterpret and re-engineer existing molecules through new scientific paradigms.

As we enter an era where molecular design meets artificial intelligence, and where the boundaries between pharmacology, biotechnology, and materials science blur, metformin reminds us that genuine progress often comes from rethinking the familiar. Old drugs can indeed teach new science—if we have the curiosity to look at them with fresh pharmaceutical eyes.

## Figures and Tables

**Table 1:** Overview of marketed metformin formulations and their key pharmaceutic characteristics.

Product Type	Release Mechanism/Technology	Dosing Frequency	Pharmaceutic Considerations
Immediate-release	Conventional tablet; rapid disintegration	2–3 times daily	High solubility but low permeability (BCS III); short intestinal window; risk of GI intolerance.
Extended-release (XR/SR/ER)	Hydrophilic polymer matrices; gel-forming systems	Once daily	Smoother plasma profile; reduced GI side effects; requires hydration-controlled release.
Osmotic-controlled release	Osmotically driven drug release via semipermeable membrane	Once daily	Designed for sustained release independent of GI pH; larger tablet size.
Gastro-retentive systems	Gel-expansion or polymer-swelling technologies	Once daily	Prolongs gastric residence time to enhance proximal gut absorption where transporters are abundant.
Delayed-release (DR)	Enteric coating dissolving at higher pH	Once daily	Minimizes systemic exposure; targets distal-gut mechanisms; beneficial in renal impairment.
Fixed-dose combinations	Co-formulated IR or XR tablets depending on partner drug	Once or twice daily	Requires compatibility of dissolution profiles; improves adherence; biopharmaceutic complexity depends on co-formulated agent.

**Table 2:** The coordinated activity of uptake transporters (organic cation transporters, OCTs, and plasma membrane monoamine transporter, PMAT) and efflux transporters (multidrug and toxin extrusion proteins, MATEs) governs metformin’s tissue-specific exposure, therapeutic variability, and safety profile. Integration of transporter distribution, regulatory mechanisms, and functional consequences provides a framework for formulation design, dose optimization, and precision pharmaceutics. OCT organic cation transporter; PMAT plasma membrane monoamine transporter; MATE multidrug and toxin extrusion protein; GI gastrointestinal; CKD chronic kidney disease; CNS, central nervous system.

Transporter	Tissue Distribution	Function	Regulatory Mechanisms	Impact on Pharmacology	Translational/Clinical Implication	Formulation Relevance
OCT1 (SLC22A1)	Liver, intestine	Uptake	Genetic polymorphisms, epigenetic control	Determines hepatic accumulation and efficacy	Determines glucose-lowering efficacy; biomarker of response	Release rate and targeting influence hepatic delivery
OCT2 (SLC22A2)	Kidney (basolateral)	Uptake	Genetic polymorphisms	Affects renal clearance	Influences clearance and nephrotoxicity risk	Dose and formulation adjustment
OCT3 (SLC22A3)	Broad (intestine, heart, tumor cells)	Uptake	Epigenetic and transcriptional control	Variable tissue-specific uptake	Explains heterogeneous tumor response	Prolonged luminal exposure; tissue targeting
PMAT (SLC29A4)	Intestine, brain	Uptake	pH-sensitive transport	Influences absorption and CNS distribution	Affects GI tolerance and CNS exposure	Delayed-release, gut-targeted systems
MATE1 (SLC47A1)	Kidney, liver (apical)	Efflux	Transcriptional and environmental regulation (pH, hypoxia)	Controls intracellular exposure	Controls systemic exposure	Formulation/dose modulation
MATE2-K (SLC47A2)	Kidney	Efflux	Genetic variants	Modifies systemic clearance	Risk of accumulation in CKD	Personalized dosing strategies

## Data Availability

No dataset was generated for the publication of this article.

## References

[1] Chaudhary S., Kulkarni A. (2024). Metformin: Past, Present, and Future. Curr. Diabetes Rep..

[2] Solini A., Tricò D. (2024). Clinical Efficacy and Cost-Effectiveness of Metformin in Different Patient Populations: A Narrative Review of Real-World Evidence. Diabetes Obes. Metab..

[3] National Center for Biotechnology Information PubChem Compound Summary for CID 4091, Metformin. https://pubchem.ncbi.nlm.nih.gov/compound/Metformin.

[4] He Z., Teng F., Yang Y., Guo R., Wu M., Han F., Tian H., Wang J., Hu Y., Jiang Y. (2025). Hydrophilic metformin and hydrophobic biguanides inhibit mitochondrial complex I by distinct mechanisms. Nat. Struct. Mol. Biol..

[5] Sharma S., Zhang Y., Akter K.A., Nozohouri S., Archie S.R., Patel D., Villalba H., Abbruscato T. (2023). Permeability of Metformin across an In Vitro Blood–Brain Barrier Model during Normoxia and Oxygen-Glucose Deprivation Conditions: Role of Organic Cation Transporters (Octs). Pharmaceutics.

[6] Farahani A., Farahani A., Kashfi K., Ghasemi A. (2025). Inhibition of hepatic gluconeogenesis in type 2 diabetes by metformin: Complementary role of nitric oxide. Med. Gas Res..

[7] Hasanvand A. (2022). The role of AMPK-dependent pathways in cellular and molecular mechanisms of metformin: A new perspective for treatment and prevention of diseases. Inflammopharmacology.

[8] He L. (2020). Metformin and systemic metabolism. Trends Pharmacol. Sci..

[9] Sakaguchi K., Sugawara K., Hosokawa Y., Ito J., Morita Y., Mizuma H., Watanabe Y., Kimura Y., Aburaya S., Takahashi M. (2025). Metformin-regulated glucose flux from the circulation to the intestinal lumen. Commun. Med..

[10] Rzeczycki P., Pęciak O., Plust M., Droździk M. (2025). Gut Microbiota in the Regulation of Intestinal Drug Transporters: Molecular Mechanisms and Pharmacokinetic Implications. Int. J. Mol. Sci..

[11] Werner E., Bell J. (1922). The Preparation of Methylguanidine, and of ββ-Dimethylguanidine by the Interaction of Dicyandiamide, and Methylammonium and Dimethylammonium Chlorides Respectively. J. Chem. Soc. Trans..

[12] Hadden D.R. (2005). Goat’s rue–French lilac–Italian fitch–Spanish sainfoin: *Galega officinalis* and metformin: The Edinburgh connection. J. R. Coll. Phys. Edinb..

[13] Bailey C.J. (2017). Metformin: Historical overview. Diabetologia.

[14] Sterne J. (1957). Du nouveau dans les antidiabétiques. La NN diméthylamino guanyl guanidine (N.N.D.G.). Maroc Med..

[15] Sulong N.A., Lee V.S., Fei C.C., Johan M.R. (2025). Exploring Multifaceted Roles of Metformin in Therapeutic Applications, Mechanistic Insights, and Innovations in Drug Delivery Systems Across Biological Contexts: A Systematic Review. Drug Deliv. Transl. Res..

[16] Dutta S., Shah R.B., Singhal S., Dutta S.B., Bansal S., Sinha S., Haque M. (2023). Metformin: A Review of Potential Mechanism and Therapeutic Utility Beyond Diabetes. Drug Des. Dev. Ther..

[17] Markowicz-Piasecka M., Huttunen K.M., Mateusiak L., Mikiciuk-Olasik E., Sikora J. (2017). Is Metformin a Perfect Drug? Updates in Pharmacokinetics and Pharmacodynamics. Curr. Pharm. Des..

[18] U.S. Food and Drug Administration (FDA) (2017). Glucophage^®^ (Metformin Hydrochloride) Tablets and Glucophage XR^®^ (Metformin Hydrochloride Extended-Release Tablets): Prescribing Information.

[19] Graham G.G., Punt J., Arora M., Day R.O., Doogue M.P., Duong J.K., Furlong T.J., Greenfield J.R., Greenup L.C., Kirkpatrick C.M. (2011). Clinical pharmacokinetics of metformin. Clin. Pharmacokinet..

[20] Tulipano G. (2021). Integrated or Independent Actions of Metformin in Target Tissues Underlying Its Current Use and New Possible Applications in the Endocrine and Metabolic Disorder Area. Int. J. Mol. Sci..

[21] Gong L., Goswami S., Giacomini K.M., Altman R.B., Klein T.E. (2012). Metformin Pathways: Pharmacokinetics and Pharmacodynamics. Pharmacogenet. Genom..

[22] Metry M., Shu Y., Abrahamsson B., Cristofoletti R., Dressman J.B., Groot D.W., Parr A., Langguth P., Shah V.P., Tajiri T. (2021). Biowaiver Monographs for Immediate-Release Solid Oral Dosage Forms: Metformin Hydrochloride. J. Pharm. Sci..

[23] Maruthur N.M., Tseng E., Hutfless S., Wilson L.M., Suarez-Cuervo C., Berger Z., Chu Y., Iyoha E., Segal J.B., Bolen S. (2016). Diabetes medications as monotherapy or metformin-based combination therapy for type 2 diabetes: A systematic review and meta-analysis. Ann. Intern. Med..

[24] Wu C.Y., Benet L.Z. (2005). Predicting drug disposition via application of BCS: Transport/absorption/elimination interplay and development of a biopharmaceutics drug disposition classification system. Pharm. Res..

[25] Streubel A., Siepmann J., Bodmeier R. (2006). Gastroretentive drug delivery systems. Expert Opin. Drug Deliv..

[26] Roy H., Brahma C.K., Nandi S., Parida K.R. (2013). Formulation and design of sustained release matrix tablets of metformin hydrochloride: Influence of hypromellose and polyacrylate polymers. Int. J. Appl. Basic Med. Res..

[27] Szymczak-Pajor I., Drzewoski J., Kozłowska M., Krekora J., Śliwińska A. (2025). The Gut Microbiota-Related Antihyperglycemic Effect of Metformin. Pharmaceuticals.

[28] Kang C.W., Nam J.H., Oh J.H., Wang E.K., Lee S.H., Shin H.J., Kim Y.B., Lee E.J., Lim B.K., Fang S. (2025). Novel mechanism whereby metformin improves glucose homeostasis: TXNIP-GLUT1 axis modulation enhances intestinal glucotonic effects. Exp. Mol. Med..

[29] González-Casanova J.E., Navarro-Marquez M., Saez-Tamayo T., Angarita L., Durán-Agüero S., Fuentes-Barría H., Bermúdez V., Rojas-Gómez D.M. (2025). New perspectives on the molecular action of metformin in the context of cellular transduction and adipogenesis. Int. J. Mol. Sci..

[30] Lin H.Y., Lu W., He Y., Fu Y., Kaneko K., Huang P., De la Puente-Gomez A.B., Wang C., Yang Y., Li F. (2025). Low-dose metformin requires brain Rap1 for its antidiabetic action. Sci. Adv..

[31] Mueller N.T., Differding M.K., Zhang M., Maruthur N.M., Juraschek S.P., Miller E.R., Appel L.J., Yeh H.C. (2021). Metformin Affects Gut Microbiome Composition and Function and Circulating Short-Chain Fatty Acids: A Randomized Trial. Diabetes Care.

[32] Jyoti D., Dey P. (2025). Mechanisms and implications of the gut microbial modulation of intestinal metabolic processes. NPJ Metab. Health Dis..

[33] Shahwar D., Baqai S., Khan F., Khan M.I., Javaid S., Hameed A., Raza A., Saleem Uddin S., Hazrat H., Rahman M.H. (2024). Proteomic Analysis of Rap1A GTPase Signaling-Deficient C57BL/6 Mouse Pancreas and Functional Studies Identify an Essential Role of Rap1A in Pancreas Physiology. Int. J. Mol. Sci..

[34] Cherra S.J., Lamb R. (2024). Interactions between Ras and Rap signaling pathways during neurodevelopment in health and disease. Front. Mol. Neurosci..

[35] Lee S.H., Bajracharya R., Min J.Y., Han J.-W., Park B.J., Han H.-K. (2020). Strategic Approaches for Colon Targeted Drug Delivery: An Overview of Recent Advancements. Pharmaceutics.

[36] Fujita Y., Inagaki N. (2016). Metformin: Clinical Topics and New Mechanisms of Action. Diabetol. Int..

[37] Shirasaka Y., Seki M., Hatakeyama M., Kurokawa Y., Uchiyama H., Takemura M., Yasugi Y., Kishimoto H., Tamai I., Wang J. (2022). Multiple transport mechanisms involved in the intestinal absorption of metformin: Impact on the nonlinear absorption kinetics. J. Pharm. Sci..

[38] Nishii R., Xue Y., Huo R., Chen J., Shen H., Chen Y., Ogasawara K. (2025). Evaluating the utility of endogenous OCT2 and MATE1/2-K biomarkers for DDI assessment in early clinical settings. J. Pharm. Sci..

[39] Cheng M., Ren L., Jia X., Wang J., Cong B. (2024). Understanding the action mechanisms of metformin in the gastrointestinal tract. Front. Pharmacol..

[40] Shin N.R., Lee J.C., Lee H.Y., Kim M.S., Whon T.W., Lee M.S., Bae J.W. (2014). An increase in the *Akkermansia* spp. population induced by metformin treatment improves glucose homeostasis in diet-induced obese mice. Gut.

[41] Wu H., Esteve E., Tremaroli V., Khan M.T., Caesar R., Mannerås-Holm L., Ståhlman M., Olsson L.M., Serino M., Planas-Fèlix M. (2017). Metformin alters the gut microbiome of individuals with treatment-naive type 2 diabetes, contributing to the therapeutic effects of the drug. Nat. Med..

[42] Zmysłowski A., Książek I., Szterk A. (2020). N-Nitrosodimethylamine contamination in the metformin finished products. Molecules.

[43] Schlingemann J., Boucley C., Hickert S., Bourasseau L., Walker M., Celdran C., Chemarin T., Pegues C., Fritzsche M., Keitel J. (2022). Avoiding N-nitrosodimethylamine formation in metformin pharmaceuticals by limiting dimethylamine and nitrite. Int. J. Pharm..

[44] Dharani S., Mohamed E.M., Rahman Z., Khan M.A. (2024). Patient in-use stability testing of FDA-approved metformin combination products for N-nitrosamine impurity. AAPS PharmSciTech.

[45] U.S. Food and Drug Administration (FDA) (2020). Control of Nitrosamine Impurities in Human Drugs—Guidance for Industry.

[46] European Medicines Agency (EMA) (2020). Scientific Review of the Risk of Nitrosamine Impurities in Human Medicines.

[47] World Intellectual Property Organization (2019). Improved Process for the Preparation of Metformin Hydrochloride. WIPO Patent.

[48] Tarry-Adkins J.L., Grant I.D., Ozanne S.E., Reynolds R.M., Aiken C.E. (2021). Efficacy and Side Effect Profile of Different Formulations of Metformin: A Systematic Review and Meta-Analysis. Diabetes Ther..

[49] Wadher K.J., Kakde R.B., Umekar M.J. (2011). Study on Sustained-Release Metformin Hydrochloride from Matrix Tablet: Influence of Hydrophilic Polymers and In Vitro Evaluation. Int. J. Pharm. Investig..

[50] Cirilli M., Moutaharrik S., Palugan L., Coldani M.E., Buscarini A., Bruni G., Gazzaniga A., Maroni A., Foppoli A., Cerea M. (2025). Gastroretentive Systems for Prolonged Release of Metformin Based on Osmotically Driven Expansion. Int. J. Pharm..

[51] Kotha A.A., Ahmad S.U., Dewan I., Bhuiyan M.A., Rahman F.I., Naina Mohamed I., Reza M.S. (2023). Metformin Hydrochloride Loaded Mucoadhesive Microspheres and Nanoparticles for Anti-Hyperglycemic and Anticancer Effects Using Factorial Experimental Design. Drug Des. Dev. Ther..

[52] Mall J., Naseem N., Haider M.F., Rahman M.A., Khan S., Siddiqui S.N. (2024). Nanostructured Lipid Carriers as a Drug Delivery System: A Comprehensive Review with Therapeutic Applications. Intell. Pharm..

[53] Abd-El Hafeez S.I., Eleraky N.E., Hafez E., Abouelmagd S.A. (2022). Design and Optimization of Metformin Hydrophobic Ion Pairs for Efficient Encapsulation in Polymeric Drug Carriers. Sci. Rep..

[54] Tsolaki E., McCartney F., Healy A.M., Brayden D.J., Ferguson S. (2025). Solidified Ionic Liquid-Based Formulations of Metformin with Enhanced GI Epithelial Permeability. Int. J. Pharm..

[55] Liu Y., Liang Y., Yuhong J., Xin P., Han J.L., Du Y., Yu X., Zhu R., Zhang M., Chen W. (2024). Advances in Nanotechnology for Enhancing the Solubility and Bioavailability of Poorly Soluble Drugs. Drug Des. Dev. Ther..

[56] Wen C., Tang J., Cao L., Fan M., Lin X., Liu G., Liang L., Liu X., Zhang J., Li Y. (2025). Strategic Approaches for Co-Encapsulation of Bioactive Compounds: Technological Advances and Mechanistic Insight. Foods.

[57] Caturano A., Nilo R., Nilo D., Russo V., Santonastaso E., Galiero R., Rinaldi L., Monda M., Sardu C., Marfella R. (2024). Advances in Nanomedicine for Precision Insulin Delivery. Pharmaceuticals.

[58] Galiero R., Caturano A., Vetrano E., Monda M., Marfella R., Sardu C., Salvatore T., Rinaldi L., Sasso F.C. (2023). Precision Medicine in Type 2 Diabetes Mellitus: Utility and Limitations. Diabetes Metab. Syndr. Obes..

[59] Naja K., Anwardeen N., Al-Hariri M., Al Thani A.A., Elrayess M.A. (2023). Pharmacometabolomic Approach to Investigate the Response to Metformin in Patients with Type 2 Diabetes: A Cross-Sectional Study. Biomedicines.

[60] Caturano A., Galiero R., Pafundi P.C. (2019). Metformin for Type 2 Diabetes. JAMA.

[61] AlKreathy H.M., Alzahrani A.A., Esmat A., Damanhouri Z.A. (2024). Effects of Genetic Variants of Organic Cation Transporters on Metformin Response in Newly Diagnosed Patients with Type 2 Diabetes. Medicine.

[62] Stocker S.L., Morrissey K.M., Yee S.W., Castro R.A., Xu L., Dahlin A., Ramirez A.H., Roden D.M., Wilke R.A., McCarty C.A. (2013). The Effect of Novel Promoter Variants in MATE1 and MATE2 on the Pharmacokinetics and Pharmacodynamics of Metformin. Clin. Pharmacol. Ther..

[63] Anwardeen N.R., Naja K., Elrayess M.A. (2024). Advancements in precision medicine: Multi-omics approach for tailored metformin treatment in type 2 diabetes. Front. Pharmacol..

[64] García-García I., Seco-Meseguer E., Borobia A.M., Carcas-Sansuán A.J. (2024). Implementing pharmacogenetics in clinical trials: Considerations about current methodological, ethical, and regulatory challenges. Expert Rev. Clin. Pharmacol..

[65] Chan P., Shao L., Tomlinson B., Zhang Y., Liu Z.M. (2018). Metformin transporter pharmacogenomics: Insights into drug disposition—Where are we now?. Expert Opin. Drug Metab. Toxicol..

[66] Caspar S.M., Schneider T., Meienberg J., Matyas G. (2020). Added Value of Clinical Sequencing: WGS-Based Profiling of Pharmacogenes. Int. J. Mol. Sci..

[67] Stillhart C., Vučićević K., Augustijns P., Basit A.W., Batchelor H., Flanagan T.R., Gesquiere I., Greupink R., Keszthelyi D., Koskinen M. (2020). Impact of Gastrointestinal Physiology on Drug Absorption in Special Populations—An UNGAP Review. Eur. J. Pharm. Sci..

[68] Chen A., Yarmush M.L., Maguire T. (2012). Physiologically Based Pharmacokinetic Models: Integration of In Silico Approaches with Micro Cell Culture Analogues. Curr. Drug Metab..

[69] Fu C., Chen Q. (2025). The Future of Pharmaceuticals: Artificial Intelligence in Drug Discovery and Development. J. Pharm. Anal..

[70] Yang K., Gonzalez D., Woodhead J.L., Bhargava P., Ramanathan M. (2025). Leveraging *In Silico* and Artificial Intelligence Models to Advance Drug Disposition and Response Predictions across the Lifespan. Clin. Transl. Sci..

[71] Ozbek O., Genc D.E., Ulgen K.O. (2024). Advances in Physiologically Based Pharmacokinetic (PBPK) Modeling of Nanomaterials. ACS Pharmacol. Transl. Sci..

[72] UK Prospective Diabetes Study (UKPDS) Group (1998). Effect of Intensive Blood-Glucose Control with Metformin on Complications in Overweight Patients with Type 2 Diabetes. Lancet.

[73] Holman R.R., Paul S.K., Bethel M.A., Matthews D.R., Neil H.A. (2008). 10-year follow-up of intensive glucose control in type 2 diabetes. N. Engl. J. Med..

[74] O’Connor L., Bailey-Whyte M., Bhattacharya M., Butera G., Hardell K.N.L., Seidenberg A.B., Castle P.E., Loomans-Kropp H.A. (2024). Association of Metformin Use and Cancer Incidence: A Systematic Review and Meta-Analysis. J. Natl. Cancer Inst..

[75] Rehman A., Satyam S.M., El-Tanani M., Prabhakar S., Kumari R., Shetty P., Mohammed S.S.N., Nafees Z., Alomar B. (2025). Metformin Beyond Diabetes: A Precision Gerotherapeutic and Immunometabolic Adjuvant for Aging and Cancer. Cancers.

[76] Campbell J.M., Stephenson M.D., de Courten B., Chapman I., Bellman S.M., Aromataris E. (2018). Metformin Use Associated with Reduced Risk of Dementia in Patients with Diabetes: A Systematic Review and Meta-Analysis. J. Alzheimers Dis..

[77] Agius L., Ford B.E., Chachra S.S. (2020). The Metformin Mechanism on Gluconeogenesis and AMPK Activation: The Metabolite Perspective. Int. J. Mol. Sci..

[78] Salvatore T., Pafundi P.C., Morgillo F., Di Liello R., Galiero R., Nevola R., Marfella R., Monaco L., Rinaldi L., Adinolfi L.E. (2020). Metformin: An Old Drug against Old Age and Associated Morbidities. Diabetes Res. Clin. Pract..

[79] Marx N., Federici M., Schütt K., Müller-Wieland D., Ajjan R.A., Antunes M.J., Christodorescu R.M., Crawford C., Di Angelantonio E., Eliasson B. (2023). 2023 ESC Guidelines for the management of cardiovascular disease in patients with diabetes. Eur. Heart J..

[80] Nevola R., Alfano M., Pafundi P.C., Brin C., Gragnano F., Calabrò P., Adinolfi L.E., Rinaldi L., Sasso F.C., Caturano A. (2022). Cardiorenal impact of SGLT-2 inhibitors: A conceptual revolution in the management of type 2 diabetes, heart failure and chronic kidney disease. Rev. Cardiovasc. Med..

[81] Palmiero G., Cesaro A., Galiero R., Loffredo G., Caturano A., Vetrano E., Rinaldi L., Salvatore T., Ruggiero R., Rosaria Di Palo M. (2023). Impact of gliflozins on cardiac remodeling in patients with type 2 diabetes mellitus & reduced ejection fraction heart failure: A pilot prospective study. GLISCAR study. Diabetes Res. Clin. Pract..

[82] Muskiet M.H.A., Tonneijck L., Smits M.M., van Baar M.J.B., Kramer M.H.H., Hoorn E.J., Joles J.A., van Raalte D.H. (2017). GLP-1 and the kidney: From physiology to pharmacology and outcomes in diabetes. Nat. Rev. Nephrol..

[83] Nabrdalik K., Hendel M., Irlik K., Kwiendacz H., Łoniewski I., Bucci T., Alam U., Lip G.Y.H., Gumprecht J., Skonieczna-Żydecka K. (2024). Gastrointestinal adverse events of metformin treatment in patients with type 2 diabetes mellitus: A systematic review and meta-analysis with meta-regression of observational studies. BMC Endocr. Disord..

[84] Infante M., Leoni M., Caprio M., Fabbri A. (2021). Long-term metformin therapy and vitamin B12 deficiency: An association to bear in mind. World J. Diabetes.

[85] Brown J.B., Conner C., Nichols G.A. (2010). Secondary failure of metformin monotherapy in clinical practice. Diabetes Care.

[86] Crowley M.J., Diamantidis C.J., McDuffie J.R., Cameron B., Stanifer J., Mock C.K., Kosinski A., Wang X., Tang S., Williams J.W. (2016). Metformin use in patients with historical contraindications or precautions. APPENDIX A, FDA Safety Announcements for Metformin.

[87] See K.C. (2024). Metformin-Associated Lactic Acidosis: A Mini Review of Pathophysiology, Diagnosis and Management in Critically Ill Patients. World J. Diabetes.

[88] Rivera D., Onisko N., Cao J.D., Koyfman A., Long B. (2023). High-Risk and Low-Prevalence Diseases: Metformin Toxicities. Am. J. Emerg. Med..

[89] Groth O., Roider G., Graw M. (2024). Medikamenteninduzierte Todesfälle bei Niereninsuffizienz [Fatalities Associated with Failure to Adjust Drug Dose in Patients with Renal Insufficiency: A Retrospective Study at the Institute of Forensic Medicine in Munich (2019–2023)]. MMW Fortschritte Med..

[90] Mariano F., Biancone L. (2021). Metformin, Chronic Nephropathy and Lactic Acidosis: A Multi-Faceted Issue for the Nephrologist. J. Nephrol..

[91] Davies M.J., Aroda V.R., Collins B.S., Gabbay R.A., Green J., Maruthur N.M., Rosas S.E., Del Prato S., Mathieu C., Mingrone G. (2022). Management of Hyperglycemia in Type 2 Diabetes, 2022: A Consensus Report by the American Diabetes Association (ADA) and the European Association for the Study of Diabetes (EASD). Diabetes Care.

[92] Agur T., Steinmetz T., Goldman S., Zingerman B., Bielopolski D., Nesher E., Fattal I., Meisel E., Rozen-Zvi B. (2025). The Impact of Metformin on Kidney Disease Progression and Mortality in Diabetic Patients Using SGLT2 Inhibitors: A Real-World Cohort Study. Cardiovasc. Diabetol..

[93] Masouri M.M., Ebrahimi R., Noori S. (2025). An Updated Systematic Review and Meta-Analysis on the Efficacy and Safety of Metformin as Add-On Therapy to Insulin in Patients with Type 1 Diabetes. Endocrinol. Diabetes Metab..

[94] Bao J., Zhao Y., Xu X., Ling S. (2025). Advances in the Use of Metformin for Liver Disease. Curr. Med. Chem..

[95] Cichocka E., Maj-Podsiadło A., Gumprecht J. (2024). Polycystic Ovary Syndrome and Type 1 Diabetes—The Current State of Knowledge. Endokrynol. Pol..

[96] Zhou Z., Luo G., Li C., Zhang P., Chen W., Li X., Tang J., Qing L. (2023). Metformin induces M2 polarization via AMPK/PGC-1α/PPAR-γ pathway to improve peripheral nerve regeneration. Am. J. Transl. Res..

[97] Kalyanaraman B., Cheng G., Hardy M., Ouari O., Sikora A., Zielonka J., Dwinell M. (2017). Mitochondria-targeted metformins: Anti-tumour and redox signalling mechanisms. Interface Focus.

[98] Amengual-Cladera E., Morla-Barcelo P.M., Morán-Costoya A., Sastre-Serra J., Pons D.G., Valle A., Roca P., Nadal-Serrano M. (2024). Metformin: From Diabetes to Cancer—Unveiling Molecular Mechanisms and Therapeutic Strategies. Biology.

[99] Di Magno L., Di Pastena F., Bordone R., Coni S., Canettieri G. (2022). The Mechanism of Action of Biguanides: New Answers to a Complex Question. Cancers.

[100] Torunoglu S.T., Zajda A., Tampio J., Markowicz-Piasecka M., Huttunen K.M. (2023). Metformin derivatives—Researchers’ friends or foes?. Biochem. Pharmacol..

[101] Abbasi M., Heath B., McGinness L. (2024). Advances in Metformin-Delivery Systems for Diabetes and Obesity Management. Diabetes Obes. Metab..

[102] Feng J., Wang X., Ye X., Ares I., Lopez-Torres B., Martínez M., Martínez-Larrañaga M.R., Wang X., Anadón A., Martínez M.A. (2022). Mitochondria as an Important Target of Metformin: The Mechanism of Action, Toxic and Side Effects, and New Therapeutic Applications. Pharmacol. Res..

[103] Finisguerra V., Dvorakova T., Formenti M., Van Meerbeeck P., Mignion L., Gallez B., Van den Eynde B.J. (2023). Metformin improves cancer immunotherapy by directly rescuing tumor-infiltrating CD8 T lymphocytes from hypoxia-induced immunosuppression. J. Immunother. Cancer.

[104] Rashad A.A., Elshafie M.F., Mangoura S.A., Akool E.S. (2025). Modulatory effect of metformin and its transporters on immune infiltration in tumor microenvironment: A bioinformatic study with experimental validation. Discov. Oncol..

[105] Abdelmoneim M., Aboalela M.A., Naoe Y., Matsumura S., Eissa I.R., Bustos-Villalobos I., Sibal P.A., Takido Y., Kodera Y., Kasuya H. (2023). The Impact of Metformin on Tumor-Infiltrated Immune Cells: Preclinical and Clinical Studies. Int. J. Mol. Sci..

[106] Zhang X., Liu J., Zhao Z., Jiang J., Geng G. (2025). Effects of long-term metformin intake on postoperative clinicopathological characteristics in patients with invasive lung adenocarcinoma and type 2 diabetes mellitus: A retrospective analysis. PLoS ONE.

[107] Wang Y., Sun Y., Hu J., Ma H. (2024). Clinical effect of treatment with metformin for type 2 diabetes on non-small cell lung cancer patients undergoing immunotherapy: A retrospective study. Int. J. Gen. Med..

[108] Chen Y., Wang X. (2024). Advances in research on the anticancer properties and mechanisms of metformin in lung cancer. Br. J. Hosp. Med..

[109] Bhati F.K., Bhat M.K. (2024). An Anti-Neoplastic Tale of Metformin through Its Transport. Life Sci..

[110] Cai H., Zhang Y., Han T.K., Everett R.S., Thakker D.R. (2016). Cation-Selective Transporters Are Critical to the AMPK-Mediated Antiproliferative Effects of Metformin in Human Breast Cancer Cells. Int. J. Cancer.

[111] Chowdhury S., Yung E., Pintilie M., Muaddi H., Chaib S., Yeung M., Fusciello M., Sykes J., Pitcher B., Hagenkort A. (2016). MATE2 Expression Is Associated with Cancer Cell Response to Metformin. PLoS ONE.

[112] Pan C., Lee L.T.O. (2025). Membrane drug transporters in cancer: From chemoresistance mechanism to therapeutic strategies. Biochim. Biophys. Acta Rev. Cancer.

[113] Saadh M.J., Mustafa M.A., Qassem L.Y., Ghadir G.K., Alaraj M., Alubiady M.H.S., Zain Al-Abdeen S.H., Shakier H.G., Alshahrani M.Y., Zwamel A.H. (2024). Targeting hypoxic and acidic tumor microenvironment by nanoparticles: A review. J. Drug Deliv. Sci. Technol..

